# Isoconiferoside, a New Phenolic Glucoside from Seeds of *Panax ginseng*

**DOI:** 10.3390/molecules16086577

**Published:** 2011-08-04

**Authors:** Jeong Ah Kim, Jeong Hyun Son, Seo Young Yang, Young Ho Kim

**Affiliations:** College of Pharmacy, Chungnam National University, Daejeon 305-764, Korea

**Keywords:** *Panax ginseng*, phenolic glucoside, isoconiferoside, spectroscopic analysis

## Abstract

A new phenolic glucoside, isoconiferoside (**1**), was isolated from the seeds of *Panax ginseng* (Araliaceae). The structure was determined to be 9-*O*-[β-d-glucopyranosyl-(1→6)-β-d-glucopyranosyl]-*trans*-coniferyl alcohol based on spectroscopic analyses (^1^H- and ^13^C-NMR, DEPT, COSY, HMQC, and HMBC) and acid hydrolysis.

## 1. Introduction

*Panax ginseng* (Aralicaceae), an ancient and popular herbal drug used in traditional Oriental medicine, has been used as a tonic and for the treatment of various diseases [1,2]. The primary phytochemical constituents of the roots, leaves, flower buds, and fruits of *P. ginseng* are dammarane-type triterpene oligoglycosides [3]. Additionally, phenolic derivatives and polyacetylenes have been isolated from the roots of *P. ginseng* and their biological activities have been studied [4]. Most notably, glucosylated phenolic components in cultured ginseng roots have been exhibited antioxidant and radical-scavenging activities [5].

**Figure 1:**
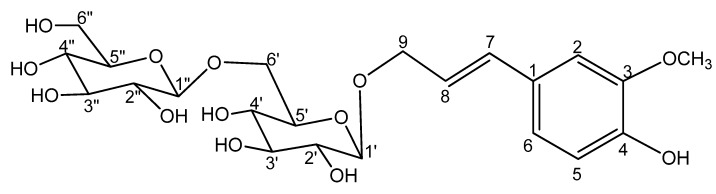
Structure of compound **1**.

As a part of ongoing research to characterize the chemical components of *P. ginseng* [2,3,4,6,7,8], seeds were collected and processed, resulting in the isolation of a new compound 9-*O*-[β-d-glucopyranosyl-(1→6)-β-d-glucopyranosyl]-*trans*-coniferyl alcohol (**1**), named isoconiferoside (Figure 1).

## 2. Results and Discussion

**Figure 2:**
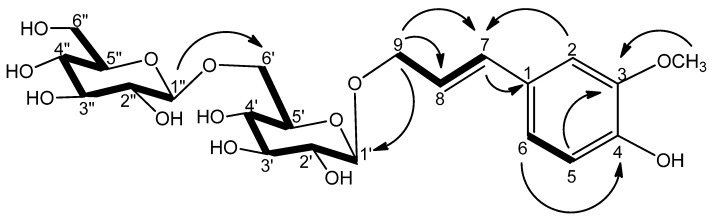
^1^H-^1^H COSY (bold lines) and key HMBC correlations (H→C) of **1**.

Compound **1** was obtained as a colorless powder with an optical rotation of −37.5° (c 0.29, MeOH). Its molecular formula (C_22_H_32_O_13_) was determined based on a peak in the HRESIMS data at *m/z* 505.2040 [M+H]^+^ (calcd for C_22_H_33_O_13_, 505.1921). Acid hydrolysis of compound **1** liberated d-glucose, identified by gas chromatographic (GC) analysis. The ^1^H-NMR spectrum of compound **1** contained signals corresponding to three aromatic protons at δ_H_ 6.99 (1H, d, *J* = 1.4 Hz, H-2), 6.84 (1H, dd, *J* = 8.3, 1.4 Hz, H-6), and 6.71 (1H, d, *J* = 8.3 Hz, H-5). The spectrum also contained two *trans*-double bond signals at δ_H_ 6.57 and 6.17 (each 1H, d, *J* = 15.8 Hz, H-7 and -8), methylene signals at δ_H_ 4.47 (1H, dd, *J* = 12.4, 5.5 Hz, H-9a) and 4.27 (1H, dd, *J* = 12.4, 6.5 Hz, H-9b), and one methoxyl resonance at δ_H_ 3.86 (3H, s). Two anomeric proton signals were observed at δ_H_ 4.40 (1H, d, *J* = 8.2 Hz, H-1″) and 4.36 (1H, d, *J* = 8.2 Hz, H-1′), suggesting that the two glucose moieties adopted β-configurations. The ^13^C-NMR and DEPT spectra of compound **1** (Table 1) exhibited 22 carbon resonances, indicating an aromatic ring [δ_C_ 149.1 (s), 147.7 (s), 130.4 (s), 121.2 (d), 116.3 (d), 110.7 (d)], an allylic group [δ_C_ 134.5 (d), 123.7 (d)], two glucopyranosyl moieties [δ_C_ 103.2 (d), 78.1 (d), 78.0 (d), 75.1 (d), 71.6 (d), 69.8 (t) and 104.9 (d), 78.1 (d), 78.0 (d), 77.1 (d), 71.6 (d), 62.8 (t)], an oxygenated methylene group [δ_C_ 71.2 (t)], and a methoxyl group [δ_C_ 56.5 (q)]. Based on a comparison of their NMR spectra compound **1** contained one more glucopyranose unit than *trans*-isoconiferin [9,10]. An HMBC experiment was conducted to determine the location of this additional glucopyranose moiety. As shown in Figure 2, the HMBC correlation between H-1″ (δ_H_ 4.40, 1H, d, *J* = 8.2 Hz) and C-6′ (δ_C_ 69.8, t) indicates that the additional glucopyranose was bound to C-6′ of the inner glucopyranose moiety. Thus, compound **1** was identified as 9-*O*-[β-d-glucopyranosyl-(1→6)-β-d-glucopyranosyl]-*trans*-coniferyl alcohol and was named isoconiferoside.

## 3. Experimental

### 3.1. General

Optical rotations were obtained using a DIP-360 digital polarimeter (Jasco, Easton, MD, USA). NMR spectra were recorded on JNM-ECA600 NMR spectrometers (JEOL Ltd., Tokyo, Japan). HRESIMS was carried out on a JMS-T100TD spectrometer (Tokyo, Japan). GC (Shimadzu-2010, Tokyo, Japan) using a DB-05 capillary column (0.5 mm i.d. × 30 m) [column temperature: 210 °C; detector temperature: 300 °C; injector temperature: 270 °C; He gas flow rate: 30 mL/min (splitting ratio: 1/20)] was used for sugar determination. Column chromatography was performed on silica gel (70–230 and 230–400 mesh, Merck) and HP-20 Diaion (Mitsubishi Chemical, Tokyo, Japan). TLC was performed on Kieselgel 60 F_254_ (1.05715; Merck, Darmstadt, Germany) or RP-18 F_254_s (Merck) plates. Spots were visualized by spraying with 10% aqueous H_2_SO_4_ solution, followed by heating.

### 3.2. Plant Material

The seeds of *P. ginseng* were collected in Geumsan province, which is well-known for ginseng cultivation in Korea, in August 2009, and were taxonomically identified by one of the authors (Young Ho Kim). Voucher specimens (CNU09105) have been deposited at the College of Pharmacy, Chungnam National University.

### 3.3. Extraction and Isolation

The powdered seeds of *P. ginseng* (4.0 kg) were extracted in MeOH (5.0 L × 3, 50 °C) and the combined extracts were concentrated *in vacuo* to dryness. The MeOH residue (202.0 g) was suspended in water (0.8 L), then partitioned with ethyl acetate (EtOAc, 0.8 L × 3), and the water layer was subjected to a Diaion HP-20 column eluted with a gradient of MeOH in H_2_O (0, 25, 50, 75, and 100% MeOH; v/v) to give six fractions, F1~F5. F3 (2.3 g) was purified on silica gel columns and eluted with CH_2_Cl_2_:MeOH:H_2_O (3.5:1:0.1, v/v) to obtain **1** (46.0 mg).

### 3.4. 9-*O*-[β-d-Glucopyranosyl-(1→6)-β-d-glucopyranosyl]-trans-coniferyl Alcohol *(**1**)*

Colorless powder, [α]${}_{D}^{16}$ -37.5 (c 0.29, MeOH) HRESIMS *m/z* 505.2040 (calcd. 505.1921 for C_22_H_33_O_13_, [M+H]^+^); For ^1^H-NMR and ^13^C-NMR spectroscopic data (in CD_3_OD), see Table 1.

### 3.5. Acid Hydrolysis and Sugars Determination of Compound ***1***

A solution of **1** (3.0 mg) in 1.0 M HCl (3.0 mL) was heated under reflux for 4 h. The reaction mixture was then concentrated to dryness under reduced pressure. The residue was extracted with EtOAc and H_2_O (5 mL, 3 times). Next, the sugar residue, obtained by concentration of the water layer, was dissolved in dry pyridine (0.1 mL) and l-cysteine methyl ester hydrochloride in pyridine (0.06 M, 0.1 mL) was added to the solution. After heating the reaction mixture at 60 °C for 2 h, 0.1 mL of trimethylsilylimidazole was added. Heating at 60 °C was continued for another 2 h, and the reaction mixture was evaporated to give a dried product, which was then partitioned between hexane and H_2_O [11]. The hexane layer was analyzed by the GC general procedure. The peak of the hydrolysate was detected at 14.12 min for D-glucose. The retention times of authentic samples (Sigma-Aldrich), after being treated in a similar manner, were 14.12 min (d-glucose), and 14.25 min (l-glucose), respectively.

## 4. Conclusions

A new phenolic glucoside, isoconiferoside (**1**), was isolated from the methanol extract of *P. ginseng* seeds. Isoconiferoside is expected to be an antioxidant and free radical scavenger and its biological activity is currently under investigation.

## Figures and Tables

**Table 1:** ^1^H and ^13^C NMR Data for compound **1** in CD_3_OD.

Positions	*δ*_C_^a,b^	DEPT	*δ*_H_^a,c^ (*J* in Hz)	HMBC (H→C)
1	130.4	C	-	-
2	110.7	CH	6.99 (d, 1.4)	4, 6, 7
3	149.1	C	-	-
4	147.7	C	-	-
5	116.3	CH	6.71 (d, 8.3)	1, 3
6	121.2	CH	6.84 (dd, 8.3, 1.4)	2, 4, 5, 7
7	134.5	CH	6.57 (d, 15.8)	1, 2, 5, 6, 9
8	123.7	CH	6.17 (d, 15.8)	1, 9
9	71.2	CH_2_	4.47 (dd, 5.5, 12.4) 4.27 (dd, 6.5, 12.4)	7, 8, 1′
1′	103.2	CH	4.36 (d, 8.2)	2′, 4′, 5′
2′	75.1	CH	3.45 (m)	3′, 5′
3′	78.0	CH	3.35 (m)^d^	2′, 4′, 5′
4′	71.6	CH	3.37 (m)^d^	3′, 5′
5′	78.1	CH	3.27 (m)^d^	2′, 3′, 4′
6′	69.8	CH_2_	4.15 (dd, 1.6, 11.7) 3.79 (dd, 5.8, 11.7)	1″, 3′, 4′, 5′
1″	104.9	CH	4.40 (d, 8.2)	6′, 3″, 5″
2″	77.1	CH	3.23 (m)	1″, 3″, 4″
3"	78.0	CH	3.35 (m) ^d^	4″, 5″
4"	71.6	CH	3.29 (m) ^d^	2″, 5″, 6″
5"	78.1	CH	3.27 (m) ^d^	3″, 4″
6″	62.8	CH_2_	3.86 (overlapped) 3.66 (dd, 5.5, 11.6)	3″, 4″, 5″
3-OCH_3_	56.5	CH_3_	3.86 (s)	3

^a^ Chemical shifts (δ) are in ppm from TMS; ^b^ Measured at 150 MHz; ^c^ Measured at 600 MHz; ^d^ Multiplicity patterns were unclear due to signal overlapping.
